# A Transcriptome Analysis Suggests Apoptosis-Related Signaling Pathways in Hemocytes of *Spodoptera litura* After Parasitization by *Microplitis bicoloratus*

**DOI:** 10.1371/journal.pone.0110967

**Published:** 2014-10-28

**Authors:** Ming Li, Zunyu Pang, Wei Xiao, Xinyi Liu, Yan Zhang, Dongshuai Yu, Minjun Yang, Yang Yang, Jiansheng Hu, Kaijun Luo

**Affiliations:** 1 School of Life Sciences, Yunnan University, Kunming, P. R. China; Key Laboratory for Animal Genetic Diversity and Evolution of High Education in Yunnan Province, Yunnan University, Kunming, P. R. China; 2 Shanghai–Ministry of Science and Technology Key Laboratory of Health and Disease Genomics, Chinese National Human Genome Center at Shanghai, Shanghai, P. R. China; Wuhan Bioengineering Institute, China

## Abstract

*Microplitis bicoloratus* parasitism induction of apoptotic DNA fragmentation of host *Spodoptera litura* hemocytes has been reported. However, how *M. bicoloratus* parasitism regulates the host signaling pathways to induce DNA fragmentation during apoptosis remains unclear. To address this question, we performed a new RNAseq-based comparative analysis of the hemocytes transcriptomes of non-parasitized and parasitized *S. litura*. We were able to assemble a total of more than 11.63 Gbp sequence, to yield 20,571 unigenes. At least six main protein families encoded by *M. bicoloratus* bracovirus are expressed in the parasitized host hemocytes: Ankyrin-repeat, Ben domain, C-type lectin, Egf-like and Mucin-like, protein tyrosine phosphatase. The analysis indicated that during DNA fragmentation and cell death, 299 genes were up-regulated and 2,441 genes were down-regulated. Data on five signaling pathways related with cell death, the gap junctions, Ca^2+^, PI3K/Akt, NF-κB, ATM/p53 revealed that CypD, which is involved in forming a Permeability Transition Pore Complex (PTPC) to alter mitochondrial membrane permeabilization (MMP), was dramatically up-regulated. The qRT-PCR also provided that the key genes for cell survival were down-regulated under *M. bicoloratus* parasitism, including those encoding Inx1, Inx2 and Inx3 of the gap junction signaling pathway, p110 subunit of the PI3K/Akt signaling pathway, and the p50 and p65 subunit of the NF-κB signaling pathway. These findings suggest that *M. bicoloratus* parasitism may regulate host mitochondria to trigger internucleosomal DNA fragmentation. This study will facilitate the identification of immunosuppression-related genes and also improves our understanding of molecular mechanisms underlying polydnavirus-parasitoid-host interaction.

## Introduction

Polydnaviruses (PDVs) have a very special life cycle. Unlike many viruses, they are not always obligate intracellular parasites, replicating inside living host cells to produce virions that can transfer genes to other cells [1]–[4]. Rather, PDVs are obligate symbionts of many endoparasitic wasps in the families Braconidae (carrying bracovirus) and Ichneumonidae (carrying ichnovirus). Both viruses have similar life cycles, wherein viral DNAs are integrated into a wasp’s genome via Wasp Integration/Excision Motif (WIM) [5] and transmitted vertically to the wasp’s offspring in a proviral form. Viruses replicate in the nucleus of the calyx cell in wasp ovaries. Mature virions are stored in the lumen of the calyx and oviduct, and the suspension of virus and protein is called calyx fluid. When a female wasp finds a host, she injects calyx fluid, venom produced by the venom gland and one or more eggs into the hemocoel of the host caterpillar. Virions infect host cells and discharge their circular dsDNA into the host nuclei, which then rapidly integrates into the host genome via the Host Integration Motif (HIM) [6]. Virulence genes are then transcribed in host cell nuclei and the cytoplasm, resulting in expression of virulence proteins. During the development of the wasp’s offspring, the host hemocoel contains innate suppressive proteins from virus gene expression. In addition, specifically among the bracoviruses, the induction of host hemocyte apoptosis causes host immunosuppression [1], [3].

Apoptosis or programmed cell death (PCD), is common to all metazoan phyla, including insects. Braconidae-induced apoptosis, however, is specifically characterized by internucleosomal DNA fragmentation. Apoptotic DNA fragmentation involves a variety of elements, including AIF, EndoG and DFF40. Every element is regulated by different signaling pathways, defined as extrinsic and intrinsic pathways. Extrinsic apoptosis pathway is triggered by the ligand-induced oligomerization of specific cell surface receptors, and this process induces the intracellular assembly of the death-inducing signaling complex for the activation of a caspase cascade initiated from caspase 8 that results in activation of caspase 3 and further cascade activation of DFF (cleavage of DFF45 releases DFF40 into the nucleus). DFF, a heterodimeric protein comprising 45 kDa and 40 kDa subunits termed ICAD/DFF45 and CAD/DFF40 [7]. The DFF complex is localized in the cellular cytoplasm, resulting in the triggering of extrinsic apoptotic stress, and activated caspase 3 cleaves DFF45 and dissociates DFF40. Caspase 7 and Granzyme B also can cleave DFF45 but with a lower efficiency than caspase 3 [8]. Activated DFF40 translocates into the nucleus. In the nucleus, the activation of DFF40 is enhanced by interaction with the chromosomal protein Histone H1 and it cleaves chromosomal DNA at internucleosomal sites into fragments of ∼200 bp. [9]–[11]. In contrast, the intrinsic pathway is also controlled by mitochondria, which collects and integrates pro- and anti-apoptotic signal stimuli from other organelles as well as from the extracellular microenvironment, such as DNA damage produced by Ataxia-Telangiectasia Mutated (ATM), endoplasmic reticulum (ER) stress and calcium overload. The intrinsic pathway can mediate caspase-independent and caspase-dependent apoptosis. Following intrinsic apoptotic stress triggering, EndoG is released from the mitochondrial intermembrane space and moves to the nucleus to produce nucleosomal DNA fragmentation, giving rise to 200∼5,000 bp sized fragments in a caspase-independent manner. AIF is another endonuclease released from the mitochondrial intermembrane space. It is a flavoprotein that produces DNA fragments up to 5,000 bp in size, and it also does not require caspase activation [12]. Releasing cytochrome *c* can also mediate cell death via activation of caspase 8, which triggers a caspase-dependent apoptosis.

Numerous viruses are well known to modulate the mitochondrial apoptosis of infected host cells by altering Mitochondrial Membrane Permeabilization (MMP) in a direct and indirect manner with viral proteins. MMP regulation is performed via the Voltage-Dependent Anion Channel (VDAC) of the outer membrane (OM), the Adenine Nucleotide Translocase (ANT) of the inner membrane (IM), and cyclophilin D (CypD) of matrix proteins. Viral proapoptotic proteins are direct inducers of MMP. They include viral protein R (Vpr), which directly interacts with ANT and VDAC, thereby triggering MMP associated with mitochondrial membrane potential (ΔΨ_m_) loss, mitochondrial intermembrane space (IMS) protein release, and caspase cascade activation. Viral proapoptotic proteins are also indirect MMP facilitators and promote apoptosis via both p53-dependent and -independent mechanisms [13]. The alteration of membrane permeability may release apoptotic-promoting factors from the mitochondria, such as AIF, EndoG, and Cyt *c* in the IMS, ultimately resulting in nuclear translocation. All of these signaling pathways involved in apoptotic DNA fragmentation are stimulated by intrinsic stress through the mitochondria via EndoG and AIF, in a process that is also called caspase-independent cell death, involving release of Cyt *c*, and extrinsic stress through caspase cascades via DFF40, which is also called caspase-dependent cell death [14].

After apoptotic stimulation, DFF40, EndoG and AIF migrate to the nucleus under the control of critical apoptosis-involved signaling pathways, including the gap junction signaling pathway, Ca^2+^ signaling pathway, PI3K/Akt signaling pathway, NF-κB signaling pathway, and ATM/p53 signaling pathway. The gap junction signaling pathway induces apoptosis via regulation of the permeability of the plasma membrane resulting in alteration of intracellular and extracellular communication via transmission of small molecules, such as apoptotic signaling ATP. Gap junction proteins are the target proteins of activated caspase 3 [15] and also Ca^2+^. The Ca^2+^ signaling pathway is involved in apoptosis via altering the permeability of the mitochondrial membrane to release apoptosis-inducing factors to trigger apoptotic caspase-dependent and -independent pathways [13]. Apoptotic caspase-dependent signaling pathways include the PI3K/Akt signaling pathway and NF-κB signaling pathway via regulation of caspase 3, and the apoptotic caspase-independent signaling pathways include regulation of the ATM/p53 signaling pathway by AIF expression [16]. The PI3K/Akt signaling pathway is crucial to many aspects of cell growth and survival, and its inhibition increases DNA fragmentation by the help of caspase 3 [17]. Baculoviruses inhibit cell apoptosis through activating the PI3K/Akt signaling pathway [18]. Nuclear Factor-κB (NF-κB) transcription factors regulate the expression of antimicrobial peptides (AMPs) and many genes involved in cell survival, such as c-IAP1/2, XIAP, and Bcl-XL. All NF-κBs are homo- or heterodimers of Rel proteins, such as p50/p65 subunits. p53 plays an important role in suppressing tumorigenesis through inducing genomic stability via DNA repair, cell cycle arrest and apoptosis. p53 promotes AIF activity and caspase-independent cell death by binding to a p53-responsive element (p53RE) in the AIF promoter, which ultimately results in efficient induction of large-scale DNA fragmentation (5 kb) [16].

In this paper, we aimed to clarify the mechanism of parasitism induction of host hemocyte apoptosis. To test the hypothesis that parasitism regulates host apoptotic signaling pathways to produce apoptotic DNA fragmentation involved in nuclear elements to the nucleus, resulting in internucleosomal DNA fragmentation from 5 kb to 200 bp, we sequenced the RNA from hemocytes of the Oriental Leafworm Moth *Spodoptera litura* parasitized by the wasp *Microplitis bicoloratus* and compared the transcriptome of hemocytes from non-parasitized controls. Using this transcription data, we obtained an overview on how *M. bicoloratus* parasitism regulates apoptosis signaling pathways during the immunosuppression and induced killing of host *S. litura* hemocytes. Furthermore, we proposed *M. bicoloratus* bracovirus products to regulate mitochondria permeability to trigger internucleosomal DNA fragmentation and block a set of key genes in the cell survival signaling pathway.

## Results

### Transcription sequencing and analysis

Gene expression profiling of *S. litura* hemocytes, both non-parasitized and parasitized, was achieved via sequencing with an Illumina Hiseq 2000 (Table S1). A million paired-end sequences (Table S2) from four samples, M1 and M2 from *S. litura* hemocytes parasitized by *M. bicoloratus* and samples S1 and S2 from non-parasitized *S. litura* hemocytes, were assembled into 3 different transcriptomes, M (M1+M2), S (S1+S2) and All (M1+M2+S1+S2), using Trinity. This gave a large number of EST cluster contigs: 15,208 (M), 15,206 (S) and 20,571 (All) (Table S3). A comparison of the transcriptome pattern of the average M and average S transcriptomes indicated that 299 consensus genes were up-regulated, and 2,441 genes were down-regulated, under *M. bicoloratus* parasitism in host hemocytes.

### *M. bicoloratus* bracovirus genes transcribed in the hemocytes of parasitized host

It is well known that polydnaviruses manipulate host cell physiology [19]. Bracoviruses encode at least 20 gene families identified from 5 species of bracoviruses, *Cotesia congregata* bracovirus (CcBV) [20], *Microplitis demolitor* bracovirus (MdBV) [21], *Glyptapanteles indiensis* bracovirus (GiBV) [22], *Glyptapanteles flavicoxis* bracovirus (GfBV) [5], and *Cotesia vestalis* bracovirus (CvBV) [23]. In the present study, genes belonging to at least 6 conserved gene families were found to be expressed in the host hemocytes parasitized by *M. bicoloratus* including 1) Ankyrin-repeat, 2) BEN domain, 3) C-type lectin, 4) Epidermal growth factor-like (EGF-like), 5) Mucin-like, and 6) protein tyrosine phosphatases (PTPs) (Table 1). Some of the proteins encoded by these genes are likely to be involved in regulating host cell death.

**Table 1:** Transcription of *M. bicoloratus* bracovirus genes during development of parasitoid *M. bicoloratus* in host hemocytes.

Protein Family	Protein	Consensus ID	Length	NCBI_E_value	NCBI_ID	Function	Species
Ankyrin-repeat	MbANK1	comp576933_c0_seq1	207	1.00E-14	ref|YP_239402.1|	viral ankyrin 1	[*Microplitis demolitor* bracovirus]
	MbANK1	comp119151_c0_seq1	558	3.00E-58	ref|YP_239402.1|	viral ankyrin 1	[*Microplitis demolito*r bracovirus]
	MbANK1	comp26305_c0_seq1	561	6.00E-40	ref|YP_239402.1|	viral ankyrin 1	[*Microplitis demolitor* bracovirus]
	MbANK2	comp728608_c0_seq1	225	6.00E-35	ref|YP_239372.1|	viral ankyrin 2	[*Microplitis demolitor* bracovirus]
	MbANK3	comp18868_c0_seq1	525	1.00E-83	ref|YP_239406.1|	viral ankyrin;	[*Microplitis demolitor* bracovirus]
Ben domain	MbBEN1	comp20976_c0_seq1	1053	4.00E-54	ref|YP_239364.1|	hypothetical protein	[*Microplitis demolitor* bracovirus]
	MbBEN1	comp20957_c0_seq1	1572	1.00E-115	ref|YP_239364.1|	hypothetical protein	[*Microplitis demolitor* bracovirus]
	MbBEN2	comp9824_c0_seq1	2046	1.00E-166	ref|YP_184800.1|	CcBV_9.1	[*Microplitis demolitor* bracovirus]
	MbBEN3	comp177162_c0_seq1	618	2.00E-72	ref|YP_184814.1|	CcBV_12.2	[*Cotesia congregata* bracovirus]
	MbBEN4	comp252441_c0_seq1	237	2.00E-34	gb|AEE09539.1|	DUF-like 1 protein	[*Cotesia congregata* bracovirus]
C-type lectin	MbCLECT1	comp19781_c0_seq1	666	1.00E-34	ref|YP_184818.1|	CcBV_2–13.1	[*Cotesia congregata* bracovirus]
	MbCLECT2	comp37160_c0_seq1	474	1.00E-43	gb|AEE09593.1|	lectin	[*Cotesia vestalis* bracovirus]
	MbCLECT3	comp375850_c0_seq1	333	1.00E-31	gb|AAS10157.1|	lectin	[*Cotesia Plutellae* Polydnavirus]
EGF-like	MbCRP1	comp22262_c0_seq1	561	8.00E-67	gb|ABB922678.1|	CRP1, egf 1.5	[*Microplitis bicoloratus* bracovirus]
Mucin-like	MbGlc1.8	comp118173_c0_seq1	153	5.69E-14	ref|YP_239419.1|	Glc1.8	[*Microplitis demolitor* bracovirus]
	MbGlc1.8	comp85587_c0_seq1	126	1.46E-54	ref|YP_239405.1|	Glc1.8	[*Microplitis demolitor* bracovirus]
PTP-like	MbPTP1	comp360492_c0_seq1	444	7.00E-72	ref|YP_239404.1|	PTP 1	[*Microplitis demolitor* bracovirus]
	MbPTP1	comp330407_c0_seq1	417	7.00E-49	ref|YP_239404.1|	PTP 1	[*Microplitis demolitor* bracovirus]
	MbPTP2	comp207973_c0_seq1	375	4.00E-64	ref|YP_239382.1|	PTP 2	[*Microplitis demolitor* bracovirus]
	MbPTP2	comp130820_c0_seq1	618	1.00E-106	ref|YP_239382.1|	PTP 2	[*Microplitis demolitor* bracovirus]
	MbPTP3	comp556935_c0_seq1	354	4.00E-59	ref|YP_239383.1|	PTP 3	[*Microplitis demolitor* bracovirus]
	MbPTP4	comp188579_c0_seq1	177	3.00E-15	ref|YP_239386.1|	PTP 4	[*Microplitis demolitor* bracovirus]
	MbPTP4	comp498102_c0_seq1	330	2.00E-33	ref|YP_239386.1|	PTP 4	[*Microplitis demolitor* bracovirus]
	MbPTP4	comp584871_c0_seq1	201	9.00E-20	ref|YP_239386.1|	PTP 4	[*Microplitis demolitor* bracovirus]
	MbPTP5	comp279111_c0_seq1	249	2.00E-11	ref|YP_239381.1|	PTP	[*Microplitis demolitor* bracovirus]
	MbPTP5	comp273967_c0_seq1	285	7.00E-35	ref|YP_239381.1|	PTP	[*Microplitis demolitor* bracovirus]
	MbPTP5	comp456541_c0_seq1	315	6.00E-38	ref|YP_239381.1|	PTP	[*Microplitis demolitor* bracovirus]
	MbPTP5	comp283025_c0_seq1	420	1.00E-41	ref|YP_239381.1|	PTP	[*Microplitis demolitor* bracovirus]
	MbPTP6	comp767898_c0_seq1	264	8.00E-42	ref|YP_239390.1|	PTP	[*Microplitis demolitor* bracovirus]
	MbPTP	comp92610_c0_seq9	252	5.90E-37	gb|ACE75309.1|	PTP	[*Glyptapanteles indiensis* bracovirus]

### Gap junction signaling pathway regulation by *M. bicoloratus* parasitism

**Figure 1:**
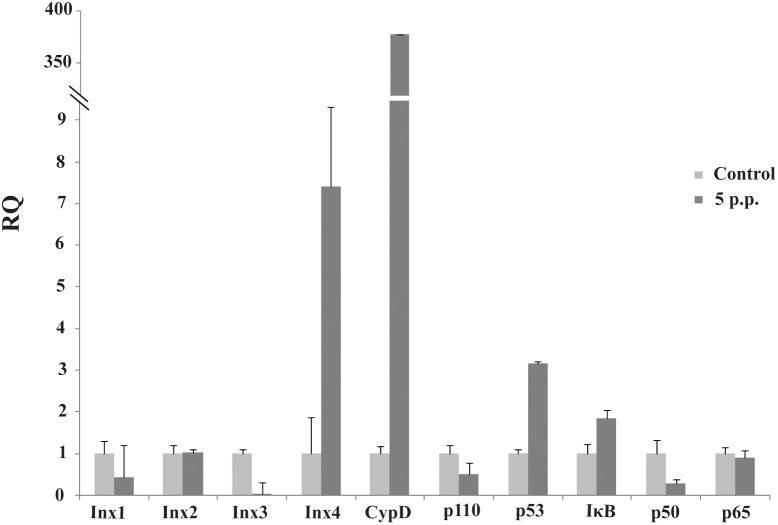
qRT-PCR detected key genes in five signaling pathways with hemocytes 5 days post-parasitization (p.p.).

Gap junction proteins form gap junction channels connecting cells for cell-cell communication and form hemichannels facilitating extracellular and intracellular communication including between ER and mitochondria to exchange small molecular, such as ATP and Ca^2+^, to trigger apoptosis [24]. In the insect circulating hemocytes, gap junction proteins form hemichannels to allow communication between the cell and environment. Under lipopolysaccharide (LPS) immunochallenge, hemichannel dye uptake decreases [25]. Typically, the decrease of the transcription level of hemichannel components and the decrease in opening of hemichannels on the cell surface result in the decrease of dye uptake. Gap junction proteins, Spli-Inx2 and Inx3, have been characterized and functioned [26] and in this study, *Spli-inx1* and *inx4* also were detected from hemocytes (Fig. S1 and Table S4). Comparisons with S and M transcriptome data indicated that all 26 elements of the gap junction signaling pathway existed in the hemocytes. During immune challenge by *M. bicoloratus* parasitization, 2 genes (Spli-GNAS, ADCY5) were not expressed in the parasitized host hemocytes. To determine the differential expression of genes, all transcriptome were assembled into a combined pool, and S1, S2, M1, and M2 were mapped using this pool to obtain reads and the RPM values of S and M. Furthermore, the analysis obtained the fold change and p-value between parasitized and non-parasitized. These analyses indicated that 12 genes (ADCY8, CPKC, GNAS, INX1, INX2, INX3, INX4, ITPR1, PKA, PLCB, PRKG, and TUBA) were down-regulated (Table 2). The qRT-PCR results indicate that the parasitization down-regulated 3 key molecules, Inx1, Inx2, Inx3, on the cell membrane, not Inx4 (Fig. 1). These molecules are involved in forming hemichannels and gap junctions, suggesting that there might be disruptions of intracellular between ER, mitochondria and extracellular molecular exchanges.

**Table 2:** The differential expression of genes regulated by *M. bicoloratus* bracovirus in the host gap junction signaling pathway.

						M/S				
A_ID	Function	read_M	RPKM_M	read_S	RPKM_S	log2(Fold_change)normalized	p-value	Result	S_ID	M-ID
comp95316_c0_seq3	adenylate cyclase 8	414	1.538851011	5365	20.66052119	−3.746951184	0	down	comp59135_c1_seq10	comp18779_c0_seq1
comp96543_c0_seq4	classical proteinkinase C	1502	6.301173776	3740	16.25542916	−1.367229143	4.6108E-232	down	comp30329_c0_seq1	comp20807_c0_seq1
comp97909_c0_seq6	guanine nucleotide-bindingprotein G(s) subunitalpha	41	0.622358814	430	6.762398457	−3.441716531	3.85048E-83	down	comp59076_c0_seq5	/
comp88846_c0_seq1	gap junction	628	3.260154915	1623	8.73E+00	−1.420902221	2.19E-107	down	comp57755_c2_seq1	comp19421_c1_seq1
comp65035_c0_seq1	gap junction	1808	16.23951848	11125	1.04E+02	−2.672414439	0.00E+00	down	comp45671_c0_seq1	comp10397_c0_seq1
comp99381_c0_seq1	gap junction	3994	22.25932132	36919	213.1714802	−3.259532924	0	down	comp59804_c0_seq1	comp30941_c0_seq1
comp121018_c0_seq1	gap junction	36	0.410377626	935	1.10E+01	−4.749973239	7.01E-217	down	comp59264_c0_seq1	comp10397_c0_seq1
comp96275_c0_seq13	inositol1,4,5-triphosphatereceptor type 1	2068	3.72067313	5214	9.718904704	−1.385230083	0	down	comp59099_c0_seq4	comp94669_c0_seq1
comp106866_c0_seq1	protein kinase A	2326	9.047590091	7695	31.01038396	−1.777145916	0	down	comp65026_c0_seq1	comp17984_c0_seq1
comp97791_c0_seq2	phosphatidylinositol phospholipase C,beta	366	2.438775601	738	5.09474476	−1.062852854	8.52689E-32	down	comp55943_c2_seq1	comp8084_c0_seq1
comp95574_c0_seq5	protein kinase,cGMP-dependent	1795	5.701522537	4620	15.20350361	−1.414984693	1.1324E-301	down	comp58204_c1_seq7	comp16873_c0_seq1
comp63482_c0_seq3	tubulin alpha	20547	224.0520545	53712	606.8022006	−1.437392362	0	down	comp41562_c0_seq1	comp14668_c0_seq1
comp94424_c0_seq3	adenylate cyclase 1	53	0.61819881	151	1.824754972	−1.561559971	1.42371E-12		comp29410_c0_seq1	comp128727_c0_seq1
comp94556_c0_seq4	adenylate cyclase 5	23	0.17008429	341	2.612558705	−3.941141659	7.22672E-73		comp55791_c2_seq2	/
comp69534_c0_seq1	adenylate cyclase 9	17	0.46329195	29	0.81880247	−0.82159384	0.069978649		comp55534_c1_seq1	comp4228_c0_seq1
comp76441_c0_seq1	cyclin-dependentkinase 1	1207	7.410567531	2282	14.51560537	−0.969948801	1.51194E-82		comp27910_c0_seq1	comp16516_c0_seq1
comp93202_c0_seq1	epidermal growthfactor receptor	6514	18.3968606	6149	17.99184785	0.032116227	0.238280263		comp72419_c0_seq1	comp33128_c0_seq1
comp83895_c0_seq2	guanine nucleotide-binding protein G(q) subunit alpha	4274	19.02930474	7980	36.81006701	−0.951877524	5.7673E-277		comp55254_c0_seq5	comp17311_c0_seq3
comp103695_c0_seq1	growth factorreceptor-bindingprotein 2	4553	31.55832529	7467	53.62135235	−0.764786958	6.7036E-179		comp59056_c4_seq1	comp101386_c0_seq1
comp112119_c0_seq1	GTPase KRas	2904	11.39420805	3605	14.6544025	−0.363033492	2.01055E-23		comp46714_c0_seq1	comp156998_c0_seq1
comp81191_c0_seq1	mitogen-activatedprotein kinasekinase 1	1619	6.495897191	2927	12.16719112	−0.905395446	3.63546E-94		comp174562_c0_seq1	comp20958_c0_seq1
comp23161_c0_seq1	mitogen-activatedprotein kinase 1/3	5679	21.44919695	7831	30.64302995	−0.514635322	3.1947E-93		comp28328_c0_seq1	comp9963_c0_seq1
comp96783_c0_seq11	son of sevenless	74	1.123281762	195	3.066669068	−1.448952634	2.40343E-14		comp47573_c1_seq1	comp17032_c0_seq1
comp97420_c0_seq2	tyrosine-protein kinase Src	126	1.148163839	460	4.342766357	−1.919285813	2.09308E-47		comp56420_c1_seq1	comp106968_c0_seq1
comp97925_c2_seq1	tight junction protein 1	71	0.575127537	454	3.810105311	−2.727877054	8.33036E-71		comp58674_c2_seq3	comp93783_c0_seq1
comp92127_c0_seq2	tubulin beta	63513	551.0471151	102657	922.7626088	−0.74378387	0		comp118372_c0_seq1	comp18397_c1_seq1

### Ca^2+^ signaling pathway regulation by *M. bicoloratus* parasitism with respect to apoptosis

Calcium ions (Ca^2+^) control every aspect of cells as cellular messengers. Ca^2+^ ions also can become death signals when delivered at physiologically aberrant conditions. Mitochondria eventually decide whether Ca^2+^ signals are life or death signals via regulation of the mitochondrial membrane proteins Bcl-2 and Bax/Bak [27]. Comparisons of the transcription data from the S and M pools indicate that all 31 elements of the Ca^2+^ signaling pathway existed in the examined hemocytes. Under *M. bicoloratus* parasitism, 3 genes (Spli-ANT, CypD, PLCG2) increased in expression, and 1 genes (Spli-PDE1) was not expressed in the parasitized hemocytes. The other 13 genes (ADCY8, ATP2A, ATP2B, CPKC, GNAS, ITPR1, ORAI1, PHKA_B, PKA, PLCB, VDAC1, VDAC2 and VDAC3) had been down-regulated (Table 3). The qRT-PCR results indicate that the parasitism up-regulated a key molecule, CypD, in the mitochondria (Fig. 1). This molecule is involved in forming a permeability transition pore complex (PTPC), suggesting that the *M. bicoloratus* alters Ca^2+^ signaling pathway to promote apoptosis.

**Table 3:** The differential expression of genes regulated by *M. bicoloratus* bracovirus in the host Ca^2+^ signaling pathway.

							M/S				
Gene family	A_ID	Function	read_M	RPKM_M	read_S	RPKM_S	log2(Fold_change) normalized	p-value	Result	S_ID	M-ID
ANT	comp95003_c0_seq1	mitochondrialadenine nucleotidetranslocator	482	5.819038231	0.5	0.006253879	9.86181365	7.35681E-80	up	/	comp41118_c0_seq1
CypD	comp93813_c0_seq1	peptidyl-prolylisomerase F(cyclophilin D)	448	6.696320619	0.5	0.007742898	9.756279236	2.02443E-75	up	/	comp11549_c0_seq1
ADCY8	comp95316_c0_seq3	adenylate cyclase 8	414	1.538851011	5365	20.66052119	−3.746951184	0	down	comp58820_c1_seq2	comp18779_c1_seq1
ATP2A	comp23165_c0_seq2	Ca2+ transportingATPase	4330	11.26751358	12042	32.46490454	−1.526711778	0	down	comp45209_c0_seq2	comp20999_c0_seq2
ATP2B	comp102625_c0_seq1	Ca2+ transportingATPase	6998	16.2324803	17020	40.90211972	−1.333292153	0	down	comp61676_c0_seq1	comp19993_c0_seq2
CPKC	comp96543_c0_seq4	classical proteinkinase C	1502	6.301173776	3740	16.25542916	−1.367229143	4.6108E-232	down	comp30329_c0_seq1	comp20807_c0_seq1
GNAS	comp97983_c1_seq2	guanine nucleotide-bindingprotein G(s) subunit alpha	149	2.139786276	363	5.400899151	−1.335732903	7.99287E-23	down	comp58416_c0_seq4	comp20437_c1_seq1
ITPR1	comp96275_c0_seq13	inositol 1,4,5-triphosphatereceptor type 1	2068	3.72067313	5214	9.718904704	−1.385230083	0	down	comp59099_c0_seq4	comp94669_c0_seq1
ORAI1	comp97095_c0_seq1	calcium release-activatedcalcium channel protein 1	238	1.972431074	588	5.048676205	−1.355930267	4.85903E-37	down	comp57934_c0_seq2	comp71014_c0_seq1
PHKA_B	comp92577_c0_seq1	phosphorylase kinasealpha/beta subunit	1191	3.399676742	3490	10.32111467	−1.602129309	6.6379E-274	down	comp58502_c0_seq5	comp101238_c0_seq1
PKA	comp106866_c0_seq1	protein kinase A	2326	9.047590091	7695	31.01038396	−1.777145916	0	down	comp65026_c0_seq1	comp17984_c0_seq1
PLCB	comp97791_c0_seq2	Phosphatidylinositolphospholipase C, beta	366	2.438775601	738	5.09474476	−1.062852854	8.52689E-32	down	comp55982_c0_seq1	comp80726_c0_seq1
VDAC1	comp90986_c0_seq1	voltage-dependentanion channel protein 1	4	0.179365731	42	1.95E+00	−3.443393109	3.25E-09	down	comp56820_c0_seq2	comp79085_c0_seq1
VDAC2	comp99405_c0_seq1	voltage-dependentanion channel protein 2	5522	67.03373029	10842	136.3583388	−1.024443805	0	down	comp60098_c0_seq1	comp23968_c0_seq1
VDAC3	comp89185_c0_seq1	voltage-dependentanion channel protein 3	2	0.093999153	26	1.27E+00	−3.751515404	1.70E-06	down	comp60098_c0_seq1	comp23968_c0_seq1
ADCY1	comp94424_c0_seq3	adenylate cyclase 1	53	0.61819881	151	1.824754972	−1.561559971	1.42371E-12		comp48930_c1_seq2	comp128727_c0_seq1
ADCY9	comp69534_c0_seq1	adenylate cyclase 9	17	0.46329195	29	0.81880247	−0.82159384	0.069978649		comp55534_c1_seq1	comp4228_c0_seq1
CALM	comp23241_c0_seq1	calmodulin	27610	270.0746841	42753	433.2707575	−0.681910456	0		comp45080_c1_seq2	comp61330_c0_seq1
CAMK2	comp97973_c0_seq1	calcium/calmodulin-dependentprotein kinase II	944	3.291051665	1737	6.273903548	−0.930814675	5.27371E-59		comp57321_c0_seq1	comp19479_c0_seq1
EGFR	comp93202_c0_seq1	epidermal growthfactor receptor	6514	18.3968606	6149	17.99184785	0.032116227	0.238280263		comp72419_c0_seq1	comp33128_c0_seq1
GNAQ	comp83895_c0_seq2	guanine nucleotide-binding protein G(q)subunit alpha	4274	19.02930474	7980	36.81006701	−0.951877524	5.7673E-277		comp50512_c0_seq1	comp166552_c0_seq1
ITPK	comp30903_c0_seq1	1D-myo-inositol-triphosphate 3-kinase	962	11.05221153	1777	21.1512899	−0.936410568	6.25084E-61		comp55786_c0_seq2	comp37996_c0_seq1
MYLK	comp95483_c0_seq1	myosin-light-chainkinase	65	1.078904349	198	3.404944919	−1.658064493	1.86308E-17		comp46122_c0_seq1	comp119788_c0_seq1
PDE1	comp96257_c0_seq5	calcium/calmodulin-dependent 3′,5′-cyclicnucleotide phosphodiesterase	8	0.058446689	531	4.019201305	−6.103643737	1.0771E-127		comp58443_c0_seq1	/
PHKG	comp97075_c0_seq1	phosphorylase kinasegamma subunit	350	2.198800775	633	4.119996628	−0.905926263	2.4877E-21		comp56788_c0_seq1	comp57668_c0_seq1
PLCG1	comp95371_c0_seq1	phosphatidylinositolphospholipase C,gamma-1	155	1.337538268	289	2.583733252	−0.949876963	3.71489E-11		comp54883_c1_seq1	comp81243_c0_seq1
PLCG2	comp94580_c0_seq1	phosphatidylinositolphospholipase C,gamma-2	110	1.770661702	159	2.651644792	−0.582598928	0.00156832		/	comp78811_c0_seq1
PPP3C	comp108295_c0_seq1	serine/threonine-proteinphosphatase 2B catalyticsubunit	2433	13.2733981	4265	24.1065111	−0.860885107	1.4074E-125		comp38261_c0_seq1	comp20495_c0_seq2
PPP3R	comp109656_c0_seq1	serine/threonine-proteinphosphatase 2B regulatorysubunit	1743	13.13252377	2743	21.41173844	−0.705257739	7.24698E-58		comp42185_c0_seq1	comp35682_c0_seq1
SPHK	comp92166_c0_seq3	sphingosine kinase	76	0.79803821	134	1.457774018	−0.869237363	3.52398E-05		comp52416_c0_seq1	comp8718_c0_seq1
STIM1	comp94633_c0_seq1	stromal interactionmolecule 1	1970	8.552321068	2535	11.40173775	−0.414865803	3.03169E-21		comp55152_c1_seq2	comp19536_c0_seq1

### PI3K/Akt signaling pathway regulation by *M. bicoloratus* parasitism

The PI3K/Akt signaling pathway is involved in multiple different pathways, including cell survival, apoptosis, cell cycle, and DNA repair, through different downstream molecules. A comparison of the transcription data from the S and M pools revealed that all 65 elements of the PI3K/Akt signaling pathway existed in the hemocytes. Under immune challenge, 4 genes (ATF4, RP-S6e, EIF4EBP1, and GNB1) were expressed in the parasitized hemocytes, and 7 genes (COL1AS, FGFR2, G6PC, p85, PPP2R3, THBS2S, and TSC1) were not expressed in the parasitized host hemocytes (Table 4). Another 19 genes (COL4A, CREB3, HSP90B, IRS1, ITGB1, LAMA3_5, LAMB1, LAMC1, PDPK1, PPP2C, PPP2R2, PPP2R5, PTEN, PTK2, RAC1, STK11, TSC2 and YWHAE) were down-regulated, (Table 4). The qRT-PCR results indicated that the parasitism down-regulated a key molecule, the p110 subunit, in the PI3K/Akt signaling pathway, suggesting that the disruption of cell survival signaling pathway by the parasitism may promote cell apoptosis (Fig. 1).

**Table 4:** The differential expression of genes regulated by *M. bicoloratus* bracovirus in the host PI3K/Akt signaling pathway.

							M/S				
Gene family	A_ID	Function	read_M	RPKM_M	read_S	RPKM_S	log2(Fold_change) normalized	p-value	Result	S_ID	M-ID
ATF4	comp93717_c0_seq1	CREB2; cyclic AMP-dependent transcription factor ATF-4	391	5.031431973	0.5	0.006665921	9.559949111	8.92893E-68	up	/	comp42406_c0_seq1
PEPCK	comp109757_c0_seq1	phosphoenolpyruvate carboxykinase (GTP)	3834	27.16382297	574	4.213334979	2.688652009	0	up	comp58069_c0_seq1	comp30301_c0_seq1
RP-S6e	comp24289_c0_seq1	small subunit ribosomal protein S6e	1353	27.7189041	0.5	0.010612644	11.35087044	8.601E-176	up	/	comp11154_c0_seq1
COL4A	comp23243_c0_seq1	type IV, alpha	33659	235.0626028	160548	1161.615892	−2.305016159	0	down	comp45047_c0_seq1	comp10045_c0_seq2
CREB3	comp101801_c0_seq1	cyclic AMP-responsive element-binding protein 3	3995	24.04913606	11685	72.87636897	−1.599466012	0	down	comp28212_c0_seq1	comp9969_c0_seq1
GSK3B	comp23136_c0_seq1	glycogen synthase kinase 3 beta	1248	13.91068253	2965	34.2400086	−1.299489856	1.9348E-170	down	comp47318_c0_seq1	comp20759_c0_seq2
HSP90B	comp103187_c0_seq1	heat shock protein 90kDa beta	3701	23.9880501	9865	66.24426184	−1.465479601	0	down	comp28148_c0_seq1	comp19950_c0_seq1
IRS1	comp97702_c0_seq2	insulin receptor substrate 1	268	2.200308994	696	5.920159847	−1.427929991	4.40292E-47	down	comp58222_c1_seq1	comp53773_c0_seq1
ITGB1	comp107868_c0_seq1	integrin beta 1	2897	11.30115561	8616	34.82213249	−1.623534251	0	down	comp46010_c0_seq2	comp20980_c0_seq2
LAMA3_5	comp99575_c0_seq1	laminin, alpha 3/5	27851	42.99292765	112433	179.8147605	−2.064340192	0	down	comp59989_c0_seq1	comp17635_c0_seq1
LAMB1	comp95243_c0_seq1	laminin, beta 1	18108	49.24188678	51196	144.2366222	−1.550479569	0	down	comp60102_c0_seq1	comp10145_c0_seq2
LAMC1	comp100060_c0_seq1	laminin, gamma 1	15999	40.61052537	52368	137.716847	−1.761779459	0	down	comp60240_c0_seq1	comp10076_c0_seq1
PDPK1	comp89371_c0_seq4	3-phosphoinositide dependent protein kinase-1	1184	5.484101314	2310	11.08513688	−1.015299457	5.13123E-90	down	comp57389_c0_seq3	comp19669_c0_seq2
PPP2C	comp99514_c0_seq1	serine/threonine-protein phosphatase 2A catalytic subunit	4007	52.05795373	8123	109.3350776	−1.07056582	0	down	comp134046_c0_seq1	comp24560_c0_seq1
PPP2R2	comp95673_c0_seq2	serine/threonine-protein phosphatase 2A regulatory subunit B	362	3.276611055	761	7.136352468	−1.122982442	1.08521E-35	down	comp56391_c0_seq2	comp21423_c0_seq2
PPP2R5	comp25110_c0_seq1	serine/threonine-protein phosphatase 2A regulatory subunit B'	414	4.316267161	1061	11.46037093	−1.408797669	9.89141E-70	down	comp226619_c0_seq1	comp18379_c0_seq1
PTEN	comp97411_c0_seq4	PTEN	233	1.759294286	911	7.126499544	−2.018196785	5.8123E-99	down	comp59211_c2_seq7	comp62639_c0_seq1
PTK2	comp89387_c1_seq2	focal adhesion kinase 1	1443	7.251236554	3747	19.50764073	−1.427740364	3.4416E-248	down	comp58747_c0_seq2	comp20857_c0_seq2
RAC1	comp102261_c0_seq1	Ras-related C3 botulinum toxin substrate 1	4703	22.53778331	13189	65.48222111	−1.538757631	0	down	comp61307_c0_seq1	comp211969_c0_seq1
STK11	comp68494_c0_seq3	serine/threonine-protein kinase 11	275	2.752794104	643	6.668487203	−1.276462805	6.66465E-37	down	comp28880_c0_seq2	comp19353_c0_seq1
TSC2	comp93326_c0_seq1	tuberous sclerosis 2	42	1.079341152	227	6.043807401	−2.48530675	1.25293E-32	down	comp31311_c0_seq1	comp76972_c0_seq1
YWHAE	comp96021_c0_seq1	14–3-3 protein epsilon	55	1.03509774	357	6.960848804	−2.749496236	2.16594E-56	down	comp58841_c0_seq1	comp18525_c1_seq1
AKT	comp103304_c0_seq1	RAC serine/threonine-protein kinase	4590	25.54854119	8713	50.24541632	−0.975751075	0		comp62555_c0_seq1	comp30027_c0_seq1
ATF2	comp63925_c0_seq1	CREBP1; cyclic AMP-dependent transcription factor ATF-2	33	1.162462472	51	1.861274773	−0.679106908	0.041341344		comp30829_c1_seq1	comp100576_c0_seq1
BRCA1	comp95658_c0_seq1	breast cancer type 1 susceptibility protein	355	2.744122154	366	2.931105739	−0.09510031	0.409730291		comp54774_c0_seq1	comp10458_c0_seq1
CCND2	comp92629_c0_seq2	cyclin D2	1830	9.174971528	2968	15.41675056	−0.748723129	2.87139E-69		comp55005_c1_seq1	comp20586_c0_seq2
CCNE	comp86772_c0_seq2	cyclin E	48	0.642601979	99	1.373128968	−1.095469805	1.62283E-05		comp29156_c0_seq1	comp130315_c0_seq1
CDC37	comp104439_c0_seq1	cell division cycle protein 37	1645	17.75049078	2932	32.77809747	−0.884873205	8.69703E-91		comp28183_c0_seq1	comp31800_c0_seq1
CDK4	comp93505_c0_seq2	cyclin-dependent kinase 4	91	1.026690365	248	2.898845626	−1.497477356	9.30914E-19		comp46659_c0_seq1	comp104770_c0_seq1
COL1AS	comp140925_c0_seq1	type I/II/III/V/XI/XXIV/XXVII, alpha	0.5	0.035296871	59	4.315126435	−6.933718735	8.23218E-15		comp89703_c0_seq1	/
EGFR	comp93202_c0_seq1	epidermal growth factor receptor	6514	18.3968606	6149	17.99184785	0.032116227	0.238280263		comp72419_c0_seq1	comp33128_c0_seq1
EIF4B	comp103484_c0_seq1	translation initiation factor 4B	3735	23.01200087	6307	40.25890145	−0.806921376	7.4266E-166		comp45352_c0_seq1	comp9966_c0_seq1
EIF4E	comp108936_c0_seq1	translation initiation factor 4E	671	7.905316174	1192	14.54950751	−0.88007525	2.69219E-37		comp38976_c0_seq1	comp18053_c0_seq1
EIF4EBP1	comp89898_c0_seq1	eukaryotic translation initiation factor 4E binding protein 1	78	2.176304201	0.5	0.01445341	7.234326533	3.10204E-18		/	comp65767_c0_seq1
FGFR2	comp86525_c0_seq1	fibroblast growth factor receptor 2	9	0.147300349	80	1.35652114	−3.203078779	1.50516E-15		comp134334_c0_seq1	/
FRAP	comp98229_c0_seq3	FKBP12-rapamycin complex-associated protein	501	1.205543964	1616	4.028672805	−1.740620375	3.1793E-143		comp57729_c1_seq1	comp19045_c1_seq1
G6PC	comp92782_c0_seq1	glucose-6-phosphatase	2	0.039148868	87	1.764346079	−5.494019182	5.9857E-22		comp51802_c0_seq1	/
GBL	comp96734_c0_seq1	G protein beta subunit-like	166	2.709305209	307	5.191149459	−0.9381311	1.47018E-11		comp45689_c0_seq2	comp52297_c0_seq1
GNB1	comp81476_c0_seq1	guanine nucleotide-binding protein G(I)/G(S)/G(T) subunit beta-1	7596	24.34298724	15821	52.52888221	−1.109604667	0		/	comp20574_c0_seq1
GNB5	comp90181_c0_seq2	guanine nucleotide-binding protein subunit beta-5	337	4.024274883	558	6.903466944	−0.778592216	6.67432E-15		comp45009_c0_seq1	comp17886_c0_seq1
GNG13	comp95223_c0_seq1	guanine nucleotide-binding protein G(I)/G(S)/G(O) subunit gamma-13	7200	46.4612554	8333	55.7102541	−0.261914759	6.81673E-29		comp61890_c0_seq1	comp24329_c0_seq1
GRB2	comp103695_c0_seq1	growth factor receptor-binding protein 2	4553	31.55832529	7467	53.62135235	−0.764786958	6.7036E-179		comp59056_c4_seq1	comp101386_c0_seq1
GYS	comp95471_c0_seq2	glycogen(starch) synthase	96	0.657627746	591	4.194417817	−2.673127505	7.16393E-90		comp55945_c1_seq1	comp231427_c0_seq1
HSP90A	comp23248_c1_seq1	molecular chaperone HtpG	30676	217.7778149	38447	282.7825966	−0.376836341	1.7963E-255		comp55995_c0_seq2	comp15914_c0_seq1
IKBKB	comp46046_c0_seq1	inhibitor of nuclear factor kappa-B kinase subunit beta	628	4.037631507	887	5.908346682	−0.549245231	6.14389E-13		comp46039_c0_seq1	comp19169_c0_seq1
INSR	comp97941_c0_seq7	insulin receptor	381	2.063839032	405	2.27290447	−0.139206596	0.201218987		comp55149_c2_seq1	comp9154_c1_seq1
JAK2	comp98009_c1_seq1	Janus kinase 2	407	1.908290622	691	3.356629968	−0.814732602	1.51786E-19		comp93258_c0_seq1	comp16460_c1_seq1
KRAS	comp112119_c0_seq1	GTPase KRas	2904	11.39420805	3605	14.6544025	−0.363033492	2.01055E-23		comp46714_c0_seq1	comp37119_c0_seq1
MAP2K1	comp81191_c0_seq1	mitogen-activated protein kinase kinase 1	1619	6.495897191	2927	12.16719112	−0.905395446	3.63546E-94		comp174562_c0_seq1	comp20958_c0_seq1
MAPK1_3	comp23161_c0_seq1	mitogen-activated protein kinase 1/3	5679	21.44919695	7831	30.64302995	−0.514635322	3.1947E-93		comp28328_c0_seq1	comp9963_c0_seq1
MYB	comp93622_c1_seq4	myb proto-oncogene protein	1093	4.733328627	1565	7.021601066	−0.568944942	2.91134E-23		comp58816_c0_seq6	comp9456_c0_seq2
MYC	comp63425_c0_seq2	Myc proto-oncogene protein	2255	7.977064372	3204	11.74260281	−0.5578224	8.82957E-45		comp47218_c0_seq2	comp20721_c0_seq2
P110	comp97931_c0_seq1	phosphatidylinositol-4,5-bisphosphate 3-kinase, PIK3C	165	1.192085437	623	4.663229594	−1.967841825	6.96353E-66		comp56297_c1_seq1	comp85699_c0_seq1
P85	comp27492_c0_seq1	phosphoinositide-3-kinase, regulatory subunit, PIK3R	0.5	0.03906187	15	1.214086421	−4.957966281	0.000103715		comp39459_c0_seq1	/
PKN;	comp67156_c0_seq1	protein kinase N	778	3.949050183	1366	7.183549165	−0.863191109	3.18306E-41		comp51044_c0_seq1	comp19192_c0_seq1
PPP2R1	comp101848_c0_seq1	serine/threonine-protein phosphatase 2A regulatory subunit A	4788	35.95160432	8113	63.11330498	−0.811888022	2.797E-215		comp28215_c0_seq1	comp10104_c0_seq1
PPP2R3	comp207060_c0_seq1	serine/threonine-protein phosphatase 2A regulatory subunit B	17	0.429343833	42	1.098957536	−1.355930267	0.000936344		comp141105_c0_seq1	/
PRKAA	comp88394_c0_seq1	AMPK; 5′-AMP-activated protein kinase, catalytic alpha subunit	935	5.517046633	1081	6.608395875	−0.260403939	9.36228E-05		comp46858_c0_seq1	comp19779_c0_seq1
RAPTOR	comp96751_c0_seq3	regulatory associated protein of mTOR	186	1.325822604	550	4.061724643	−1.615204683	1.12036E-44		comp28927_c0_seq2	comp85394_c0_seq1
RHEB	comp94584_c0_seq1	Ras homolog enriched in brain	150	2.527973388	197	3.43971752	−0.444308815	0.006072962		comp83256_c0_seq1	comp65541_c0_seq1
RPS6KB	comp110869_c0_seq1	p70 ribosomal S6 kinase	1391	8.265982996	2672	16.4505016	−0.992873274	3.286E-100		comp70041_c0_seq1	comp10389_c0_seq1
SOS	comp96783_c0_seq11	son of sevenless	74	1.123281762	195	3.066669068	−1.448952634	2.40343E-14		comp47573_c1_seq1	comp17032_c1_seq1
THBS2S	comp28902_c0_seq1	thrombospondin 2/3/4/5	31	0.720784511	15	0.361335244	0.996230029	0.029739095		comp378223_c0_seq1	/
TSC1	comp87058_c0_seq1	tuberous sclerosis 1	15	0.611757829	46	1.943665035	−1.667747046	5.20854E-05		comp30877_c0_seq1	/
YWHAB_Q_Z	comp63845_c0_seq2	14-3-3 protein beta/theta/zeta	25941	59.75452622	34171	81.54871003	-0.448614059	0		comp45161_c0_seq1	comp17512_c0_seq2

### NF-κB signaling pathway regulation by *M. bicoloratus* parasitism

The NF-κB signaling pathway regulates gene expression via regulation of nuclear transcription factor. Comparison of the transcription data from the S and M pools indicates that all 18 elements of NF-κB signaling pathway existed in the hemocytes. Under *M. bicoloratus* parasitism, 1 gene (Spli-PLCG2) was expressed in the parasitized host hemocytes, and 5 genes (Spli-CSNK2A, MYD88, P50, P65 and XIAP) were down-regulated (Table 5). The qRT-PCR results indicate that the parasitism down-regulated two key molecules, the p50 (Relish) and p65 (Dorsal) subunits in the NF-κB signaling pathway, suggesting the disruption of the cell survival signaling pathway (Fig. 1).

**Table 5:** The differential expression of genes regulated by *M. bicoloratus* bracovirus in the host NF-κB signaling pathway.

							M/S				
Gene family	A_ID	Function	read_M	RPKM_M	read_S	RPKM_S	log2(Fold_change)normalized	p-value	Result	S_ID	M-ID
CSNK2A	comp67611_c0_seq1	casein kinase II subunitalpha	1490	10.76489273	2927	21.90894546	−1.025186101	1.5073E-115	down	comp56988_c0_seq2	comp10015_c0_seq2
MYD88	comp68137_c0_seq1	myeloid differentiationprimary response proteinMyD88	577	6.026390147	1241	13.42853162	−1.155935577	4.14962E-60	down	comp76362_c0_seq1	comp49638_c0_seq1
P50	comp97501_c11_seq1	nuclear factor kappa-Bp105/100, Relish 1A	756	8.18783011	1588	1.78E+01	−1.121828459	4.83E-73	down	comp57569_c0_seq1	comp46759_c0_seq1
P65	comp89974_c0_seq4	nuclear factorkappa-B/Rel, Dorsal 1A	725	6.340266258	2092	1.90E+01	−1.579905637	2.35E-161	down	comp58671_c0_seq4	comp19997_c0_seq5
XIAP	comp66362_c0_seq1	E3 ubiquitin-proteinligase XIAP, Bcl-2	3120	19.18253441	7814	49.77372136	−1.375590912	0	down	comp62965_c0_seq1	comp17943_c0_seq1
ATM	comp98156_c1_seq4	ataxia telangiectasiamutated family protein	659	2.224231496	928	3.245023613	−0.544922026	2.64245E-13		comp56187_c0_seq2	comp18276_c0_seq1
BIRC2_3	comp98152_c0_seq6	baculoviral IAPrepeat-containing protein2/3	1252	4.419168216	1858	6.794495692	−0.620591625	8.2147E-32		comp57322_c0_seq1	comp52055_c0_seq1
CSNK2B	comp102869_c0_seq1	casein kinase II subunitbeta	2230	21.59701753	3660	36.72360578	−0.765875624	1.66408E-88		comp55287_c0_seq1	comp20218_c0_seq1
IKB	comp108698_c0_seq1	inhibitor of nuclearfactor kappa-B, Cactus	3403	15.84566756	3613	1.74E+01	−0.137465789	0.000117519		comp46039_c0_seq1	comp19169_c0_seq1
IKKB	comp46046_c0_seq1	inhibitor of nuclearfactor kappa-B kinasesubunit beta	628	4.037631507	887	5.908346682	−0.549245231	6.14389E-13		comp46039_c0_seq1	comp19169_c0_seq1
IRAK4	comp50303_c0_seq1	interleukin-1receptor-associatedkinase 4	486	4.085524166	723	6.296875769	−0.624115019	3.09261E-13		comp55403_c1_seq1	comp50008_c0_seq1
MALT1	comp86328_c0_seq1	MALT1	110	1.662564556	120	1.879067547	−0.176606568	0.386011433		comp29501_c0_seq1	comp69294_c0_seq1
MAP3K7	comp97891_c0_seq2	mitogen-activatedprotein kinase kinasekinase 7	541	3.421954775	876	5.740588546	−0.746377962	3.47289E-21		comp47143_c1_seq1	comp18892_c0_seq2
MAP3K7IP1	comp96428_c0_seq2	TAK1-binding protein 1	226	2.447684663	387	4.342434795	−0.82708648	9.53466E-12		comp27058_c0_seq1	comp60240_c0_seq1
PLCG1	comp95371_c0_seq1	phosphatidylinositolphospholipase C,gamma-1	155	1.337538268	289	2.583733252	−0.949876963	3.71489E-11		comp54883_c1_seq1	comp81243_c0_seq1
PLCG2	comp94580_c0_seq1	phosphatidylinositol phospholipase C, gamma-2	110	1.770661702	159	2.651644792	−0.582598928	0.00156832		/	comp78811_c0_seq1
TRAF6	comp91031_c0_seq1	TNF receptor-associatedfactor 6	38	0.903867364	54	1.330730718	−0.558035675	0.079258626		comp52687_c2_seq1	comp130761_c0_seq1
UBE2I	comp106025_c0_seq1	ubiquitin-conjugatingenzyme E2 I	1630	22.02296837	1997	27.95384991	−0.344038054	2.46942E-12		comp57828_c0_seq1	comp16034_c0_seq1

### ATM/p53 signaling pathway regulation by *M. bicoloratus* parasitism

The ATM/p53 signaling pathway plays an important role in cell cycle control and apoptosis. In normal cells, the p53 protein level is low. DNA damage and stress signaling may trigger an increase of p53 protein levels, which has three major functions: cell cycle arrest, DNA repair and apoptosis. The cell cycle arrest prevents replication of proteins involved in DNA repair. Apoptosis avoids proliferation of cells containing abnormal DNA. p53 is a transcriptional activator that regulates the expression of MDM2. A comparison of the transcription data from the S and M pools indicate that all 21 elements of the ATM/p53 signaling pathway existed in the hemocytes. Under *M. bicoloratus* parasitism, 1 gene (Spli-SESN), was expressed in the parasitized host hemocytes, and 1 gene (CYC) was not expressed in the parasitized host hemocytes. Another 3 genes (Spli-PPM1D, PTEN, and TSC2) were down-regulated (Table 6). The qRT-PCR results indicate that the parasitism increased expression of a key molecule, p53, in the ATM/p53 signaling pathway (Fig. 1).

**Table 6:** The differential expression of genes regulated by *M. bicoloratus* bracovirus in the host ATM/p53 signaling pathway.

							M/S				
Gene family	A_ID	Function	read_M	RPKM_M	read_S	RPKM_S	log2(Fold_change)normalized	p-value	Result	S_ID	M-ID
PPM1D	comp23378_c0_seq1	protein phosphatase 1D	436	5.195890813	968	11.95154904	−1.201754598	4.38033E-50	down	comp45971_c0_seq1	comp9608_c0_seq1
PTEN	comp97411_c0_seq4	PTEN	233	1.759294286	911	7.126499544	−2.018196785	5.8123E-99	down	comp59211_c2_seq7	comp62639_c0_seq1
TSC2	comp93326_c0_seq1	tuberous sclerosis 2	42	1.079341152	227	6.043807401	−2.48530675	1.25293E-32	down	comp31311_c0_seq1	comp12675_c0_seq1
ATM	comp98156_c1_seq4	ataxia telangiectasia mutated family protein	659	2.224231496	928	3.245023613	−0.544922026	2.64245E-13		comp56187_c0_seq2	comp18276_c0_seq1
ATR	comp85208_c0_seq1	serine/threonine-protein kinase ATR	62	1.180743424	98	1.933593758	−0.71158922	0.00305328		comp53816_c1_seq1	comp15900_c0_seq1
CCNB	comp86097_c0_seq2	cyclin B	984	4.996128296	1852	9.742149268	−0.963429564	1.7509E-66		comp57367_c0_seq1	comp19132_c0_seq2
CCND2	comp92629_c0_seq2	cyclin D2	1830	9.174971528	2968	15.41675056	−0.748723129	2.87139E-69		comp55005_c1_seq1	comp20586_c0_seq2
CCNE	comp86772_c0_seq2	cyclin E	48	0.642601979	99	1.373128968	−1.095469805	1.62283E-05		comp29156_c0_seq1	comp130315_c0_seq1
CCNG2	comp81700_c0_seq1	cyclin G2	2216	12.41316031	3124	18.1300477	−0.546512258	3.78105E-42		comp58929_c0_seq2	comp21305_c1_seq2
CDK1	comp76441_c0_seq1	cyclin-dependent kinase 1	1207	7.410567531	2282	14.51560537	−0.969948801	1.51194E-82		comp27910_c0_seq1	comp16516_c0_seq1
CDK4	comp93505_c0_seq2	cyclin-dependent kinase 4	91	1.026690365	248	2.898845626	−1.497477356	9.30914E-19		comp46659_c0_seq1	comp104770_c0_seq1
CHK2	comp93714_c0_seq1	serine/threonine-protein kinase Chk2	116	1.042448685	212	1.973821482	−0.921015145	3.88281E-08		comp29305_c0_seq1	comp185379_c0_seq1
CYC	comp93023_c0_seq1	cytochrome c	1	0.030570159	77	2.438730115	−6.317862227	1.87344E-19		comp96131_c0_seq1	/
EI24	comp94889_c0_seq2	etoposide-induced 2.4 mRNA	143	1.901385289	220	3.030624197	−0.672564063	2.18683E-05		comp87761_c0_seq1	comp67505_c0_seq1
GADD45	comp87685_c0_seq2	growth arrest and DNA-damage-inducible protein	933	10.69806017	999	11.86763536	−0.149683283	0.029048024		comp58271_c0_seq2	comp19840_c0_seq1
P53	comp63894_c0_seq1	p53	1659	10.61581334	2957	1.96E+01	−0.884896043	1.49E-91		comp27951_c0_seq1	comp20024_c0_seq1
RCHY1	comp94630_c0_seq1	RING finger and CHY zinc finger domain-containing protein 1	80	1.340540829	140	2.430487591	−0.858430608	2.87028E-05		comp30489_c0_seq1	comp128683_c0_seq1
RFWD2	comp93760_c0_seq1	E3 ubiquitin-protein ligase RFWD2	122	1.853497561	146	2.298054677	−0.310162907	0.094260381		comp99768_c0_seq1	comp10686_c1_seq1
RRM2	comp100970_c0_seq1	ribonucleoside-diphosphate reductase subunit M2	4699	44.24117718	6338	61.82281522	−0.482749578	1.81544E-67		comp61643_c0_seq1	comp10097_c0_seq1
SESN	comp313998_c0_seq1	sestrin	12	0.967587612	0.5	0.041769028	4.533886815	0.000805877		/	comp150130_c0_seq1
SIAH1	comp38385_c0_seq1	E3 ubiquitin-protein ligase SIAH1	886	4.980482149	1456	8.479580246	−0.767707437	4.40084E-36		comp189698_c0_seq1	comp20596_c1_seq1

## Discussion

*M. bicoloratus* parasitism regulated host hemocyte apoptosis, resulting in DNA fragmentation. In this study, we examined the impacts of both the apoptotic caspase-dependent and -independent signaling pathways on the host hemocytes based on transcriptome data. Our results demonstrated that bracovirus proteins are expressed in the host hemocytes, suggesting their roles in DNA fragmentation by regulating key signaling pathways, resulting in the triggering of caspase-dependent and -independent pathways.

First, we found that *M*. *bicoloratus* parasitism regulated genes involved in forming the PTPC, which control mitochondrial apoptosis. Following *M*. *bicoloratus* parasitization, Spli-CypD was dramatically up-regulated (Table 3, Fig. 1). PTPC, which is a large multiprotein structure assembled at the contact sites between outer membrane (OM) and inner membrane (IM) of mitochondria, regulates MMP. PTPC activation provokes a sudden increase in the IM permeability to solutes of low molecular weight, causing the unregulated entry of water and osmotic swelling of the mitochondrial matrix. Numerous studies suggest that the PTPC is assembled by ANT (in the IM), VDAC (in the OM) and mitochondrial matrix protein cyclophilin D (Cyp D) [13]. According to our data, under *M*. *bicoloratus* parasitism, PTPC can form in the mitochondria of host hemocytes. Some DNA viral proteins may be direct inducers of MMP, and some may be indirect MMP facilitators, resulting in the activation of the mitochondrial apoptosis pathway [13]. This suggests an inducing condition of PTPC. *M*. *bicoloratus* parasitism may promote cell death via regulation of PTPC formation to release factors involved in DNA fragmentation from mitochondria into nuclei to cleave DNA.

PTPC formed suddenly during immunochallenge, AIF, EndoG, and Cyt *c* in the mitochondria were released from the inter-mitochondrial space into the cytoplasm. EndoG and AIF directly move into the nucleus to digest DNA [28], [29]. In mammals, the endonuclease DFF40 initiates DNA fragmentation. A recent report found that in *Caenorhabditis elegans*, there is an unexpected connection between Dicer and DNA degradation during cell death [30]. The Dicer-family RNase III enzymes include helicase, PAZ, RNaseIII, and dsRNA-binding domains [30]. CED-3 cleaves DCR-1, the *C. elegans* Dicer orthologue, as a candidate, at a specific position to yield a short isoform termed tDCR-1, which lacks the helicase and PAZ domain, and gains the capacity to cleave DNA into fragments [31]. Once DNA suffers double-strand breaks, the ATM signaling pathway activates and interacts with many different proteins to induce cell cycle arrest, increase DNA repair, and inhibit apoptosis, which involves the p53 signaling pathway, NF-κB signaling pathway and PI3K/Akt signaling pathway via the activation of IKKβ and p53 [32]. Typically, the activated ATM signaling pathway should inhibit host cell apoptosis for cell survival [33], [34].

At this point, we wish to examine how the parasitism inhibited the ATM-triggered DNA repair and cell survival signaling pathways. During DNA damage in the host hemocytes, ATM is expressed (Table 6). The ATM signaling pathway is responsible for DNA repair via activation of the related cell survival signaling pathway [35]. DNA damage may activate protein kinases, such as ATM, to phosphorylate p53 at one of these three residues, which thereby increases the p53 level. After the DNA damage is repaired, the ATM kinase is no longer active. p53 will be quickly dephosphorylated and destroyed by the accumulated MDM2 [36]. p53 is conserved across eukaryotic organisms, and the decrease of transcriptional levels of genes regulated by p53 leads to a subdued resistance to pathogens infections. In *C. elegans*, p53/CEP-1 are inhibited by the nucleolar protein NOL-6, a nucleolar RNA-associated protein, causing innate immune suppression [37].

It is well known that PI3K/Akt signaling pathway regulates cell survival and apoptosis. PI3K is composed of heterodimers of inhibitory adaptor/regulatory (p85) and a catalytic (p110) subunits. p85 binds and integrates signals from various cellular proteins, including transmembrane tyrosine kinase-linked receptors and intracellular proteins, providing an integration point for activation of p110. Akt, which contains a PH domain in the N-terminal region, is the primary downstream mediator of the effects of PI3K. The PH domain of Akt interacts with 3′-phosphoinositides, contributing to recruitment of Akt to the plasma membrane. Recruitment to the membrane results in a conformation change, contributing to exposure of two crucial phosphorylation sites, serine 473 and threonine 308, for activation. An unexpected finding is that p85 was not expressed under *M. bicoloratus* parasitism (Table 4). HSV-1, herpes simplex virus, induces the phosphorylation of Akt during infection of oral epithelial cells, leading to anti-apoptosis, and inhibition of HSV-1-induced PI3K activity increases DNA fragmentation [17]. Insect baculovirus AcMNPV activates PI3K/Akt signaling pathway antiapoptosis to replicate itself in the host cell via enhancing phosphorylation of Ser 473 of Akt [18]. In our laboratory, overexpression of the gap junction proteins Inx2 and Inx3 caused dramatic apoptosis in Sf9 and Spli221 cells but no phosphorylation of Akt in Hi5 cell lines, which reveals an anti-apoptosis function [26].

NF-κB signaling pathway regulates cell survival and apoptosis. In innate immunosuppression in invertebrates, it is well known that PDV protein vankyrins, which lack the phosphorylation and ubiquitination domains, function as IκB mimics via completion for the NF-κB site with IκB [38]. This results in retention of NF-κB in the cytoplasm, which inhibits immune gene expression for products such as antimicrobial peptides (AMPs) [39]. Three vankyrin genes were expressed in the host hemocytes (Table 1). NF-κB is constituted of p50 and p65 subunits. Normally, the p50/p65 complex is released from IκB and translocated to the nucleus to activate the transcription of genes involved in cell survival. During the immunochallenge, p50 and p65 were down-regulated by *M. bicoloratus* parasitism (Table 5, Fig. 1) suggesting that *M. bicoloratus* blocked the critical signaling pathway to promote cell apoptosis.

Ca^2+^ overload from the ER to mitochondria is required for initiation of programmed cell death. An unexpected result concerns Ca^2+^ loading between the endoplasmic reticulum and mitochondria. Previously, we proposed that innexin hemichannels on the ER can be Ca^2+^ channels, providing a pannexin 3-like function in the mammal to deliver Ca^2+^ [24], [31]. In such a case, *inx* genes should be up-regulated to produce more hemichannels, but 3 *inx* genes were been down-regulated, only *inx4* was up-regulated (Table 2, Fig. 1). This suggests a disruption in hemichannel activation under *M. bicoloratus* parasitism.

In Table 1 and Fig. 1, we show six types of gene transcriptions in the parasitized host hemocytes related to the Ankyrin-repeat, PTP, C-type lectin, Ben domain, Mucin-like and EGF-like families. Recent research indicates that C-type lectin (SIGN-R1) enhances uptake and the processing of circulating apoptotic cells in the spleen [40]. CpBV-lectin encoded by *C. plutellae* bracovirus is secreted into plasma and binds to the surface of parasitoid eggs to induce host immunosuppression via inhibition of host hemocyte non-self recognition [41]. In our research system, considering the interaction between *M. bicoloratus* bracovirus proteins and apoptosis, whether MbBV-lectin provides a relative contribution to apoptotic cell clearance, similar to SIGN-R1, requires further examination. However, it is reasonable to indicate that most important genes displayed less transcription in the host hemocytes during apoptosis. The Ben domain-containing proteins are well known to be involved in the transcriptional repression through its interaction with histone deacetylase, and overexpression causes cell cycle arrest [42]. The ankyrin-repeat protein family acts as inhibitors of nuclear transcription factors via binding of NF-κB homodimers [39]. Protein tyrosine phosphatases are the largest family encoded by bracovirus, and PTPs are well known as a regulator of apoptosis in human [43], such as PTP-1B regulation of the PI3K/Akt cascade to influence the nuclear localization of FOXO1, a transcription factor that regulates the expression of several pro-apoptotic genes [44], and SHP-1 that disrupts anti-apoptotic pathways through the regulation of the p85 subunit of PI3K [45], and TC-PTP also regulates p53 expression during apoptosis [46]. PTP-H2 from MdBV is a functional tyrosine phosphatase [47] and induces apoptosis of Sf21 cells [48]. MbCrp (egf-like) disrupts the cytoskeleton of host hemocytes [49].

In conclusion, our findings demonstrated that *M. bicoloratus* parasitism could regulate critical signaling pathways of host hemocytes to promote apoptosis to suppress host cellular immunity. Bracovirus may regulate proteins to form a PTPC structure that altered mitochondrial permeability, resulting in the release of DNA fragmentation elements, causing DNA damage and keeping ATM expression. This might have implications for better understanding of the mechanism of innate immunosuppression via the apoptosis pathway. However, analysis of the bracovirus proteins regulation of the critical signaling pathway may involve three levels in the cell, as a ligand binding to receptor on the cell surface, as a mini-protein to compete with scaffold proteins, as a nuclear factor to promote gene expression, as a host translation inhibitory factor to inhibit host protein translation or utilization of an RNAi mechanism [50] to inhibit gene expression on the mRNA level. The proteins responsible for specific signaling molecules in host hemocytes remain to elucidated.

## Materials and Methods

### Insect rearing and experimental animals

The *S. litura* colony was reared on an artificial diet (formulated according to [51]) at 27±1°C, RH 60–80%, and under a 12∶12 h photoperiod regimen. The parasitoid *M. bicoloratus* colony was maintained on *S. litura* larvae reared in the laboratory according to established methods [52]. Adults were also provided with honey as a dietary supplement.

### Isolation of hemocytes from larvae of *S. litura*

Hemocytes were collected 5 days post-parasitization from parasitized *S. litura* larvae (more than 1,000) (when immature parasitoids in the host developed to the second larvae [52], approximately 21% hemocytes underwent apoptosis [1]) and named ‘M’ (parasitized by *M. bicoloratus*) in this paper. The fourth instar *S. litura* larvae were used to collect hemocytes to serve as the control group, named ‘S’ (non-parasitized *S. litura* hemocytes) in this paper.

### Total RNA extraction

Total RNA was isolated from hemocytes using an RNeasy Plus Universal Mini Kit (QIAGEN, Maryland, USA), which is specific for genome DNA elimination, according to the manufacturer’s instructions. The concentration of each RNA sample was determined by measuring OD at A260/A280 using the NanoDrop 2000 and running 1 x TBE agarose gel. High quality samples (with an A260/A280 ratio >2.0, A260/A230>2.0, concentration>500 ng/ul) showing 28S and 18S RNA bands clearly were stored at −80°C until use. RNA was prepared from at least two biological replicates and used for independent library preparations.

### Transcription mRNA sequencing, assemble, gene predicted

Sequencing libraries were prepared using a RNA-Seq sample preparation kit from Illumina following the manufacturer’s instructions. The transcription sequences were sequenced using an Illumina Hiseq2000, and the total base number was more than 26.3 Gb per sample. There were two replications for the M1, M2, S1, and S2 pools. RNA-seq de novo assembly was performed using Trinity [53]. GetORF in EBOSS were used to find protein from contigs [54].

### Gene Ontology (GO) and KEGG data

GO Slim test were assigned to the NR-annotated transcripts using a local Blast2GO pipeline *b2g4pipe* [55] with access to a local GO MySQL database (version of April 2013). The Kyoto Encyclopedia of Genes and Genomes (KEGG) was used for analysis of molecular networks [56].

### Definition of up- or down-regulated genes based on fold change

Clean reads were mapping to assembled contigs, to get RPM value based on reads number [57]. Statistical analysis of data was performed using DESeq [58]. Transcript abundances for each gene were expressed as a weighted mean of counts from each replicate normalized to the overall library size (known as ‘base mean’). *p*-values (adjusted for false discovery rate) were generated for each gene in pair-wise comparisons between different conditions. In our analyses, we used an adjusted *p*-value of 0.001 as a criteria for identifying significant differences in gene expression.

### Total RNA isolation, cDNA synthesis and qRT-PCR

Total RNA was isolated from hemocytes of parasitized *S. litura* larvae 5 days post-parasitization using RNAiso Plus (TaKaRa, Dalian, China), according to manufacturer’s instructions, including DNase treatment. The concentration and purity of each RNA sample was determined by measuring OD at A260/A280 using NanoDrop 2000. Samples with an A260/A280 ratio >2.0) were used to synthesize cDNA using Oligo d (T) 18 primers following manufacturer’s instruction (TaKaRa, Dalian, China). All cDNA samples were stored at −80°C for preservation. qRT-PCR was performed using SYBR PCR Kit (Takara, Dalin, China) with the ABI 7500 system following the cycling parameters: 50°C, 2 min; 95°C, 10 mim; 95°C, 5 sec, 60°C, 34 sec, 40 cycles; 95°C, 15 sec; 60°C, 1 min; 95°C, 30 sec; 60°C, 15 sec. The 2-ΔΔCT method was used to get the relative mRNA levels [59]. 18S rDNA gene was used as the housekeeping genes for normalization. Three replications have been carried out for per sample.

### GenBank accession numbers

The whole RNA-Seq project was deposited into DDBJ/DRA/GenBank under the accession DRA001149.

## Supporting Information

Figure S1 **Completed ORF and short qRT-PCR products.**
(TIF)

Table S1 **Sample information.**
(XLS)

Table S2 **Sequencing output and quality.**
(XLSX)

Table S3 **EST cluster contigs.**
(XLSX)

Table S4 **Primers of completed ORF and short qRT-PCR.**
(XLSX)
