# Supplementary figures and images for: A Transcriptome Analysis Suggests Apoptosis-Related Signaling Pathways in Hemocytes of Spodoptera litura After Parasitization by Microplitis bicoloratus
Source: PLoS One. 2014 Oct 28;9(10):e110967. doi: 10.1371/journal.pone.0110967 (PMC4211697; doi:10.1371/journal.pone.0110967)

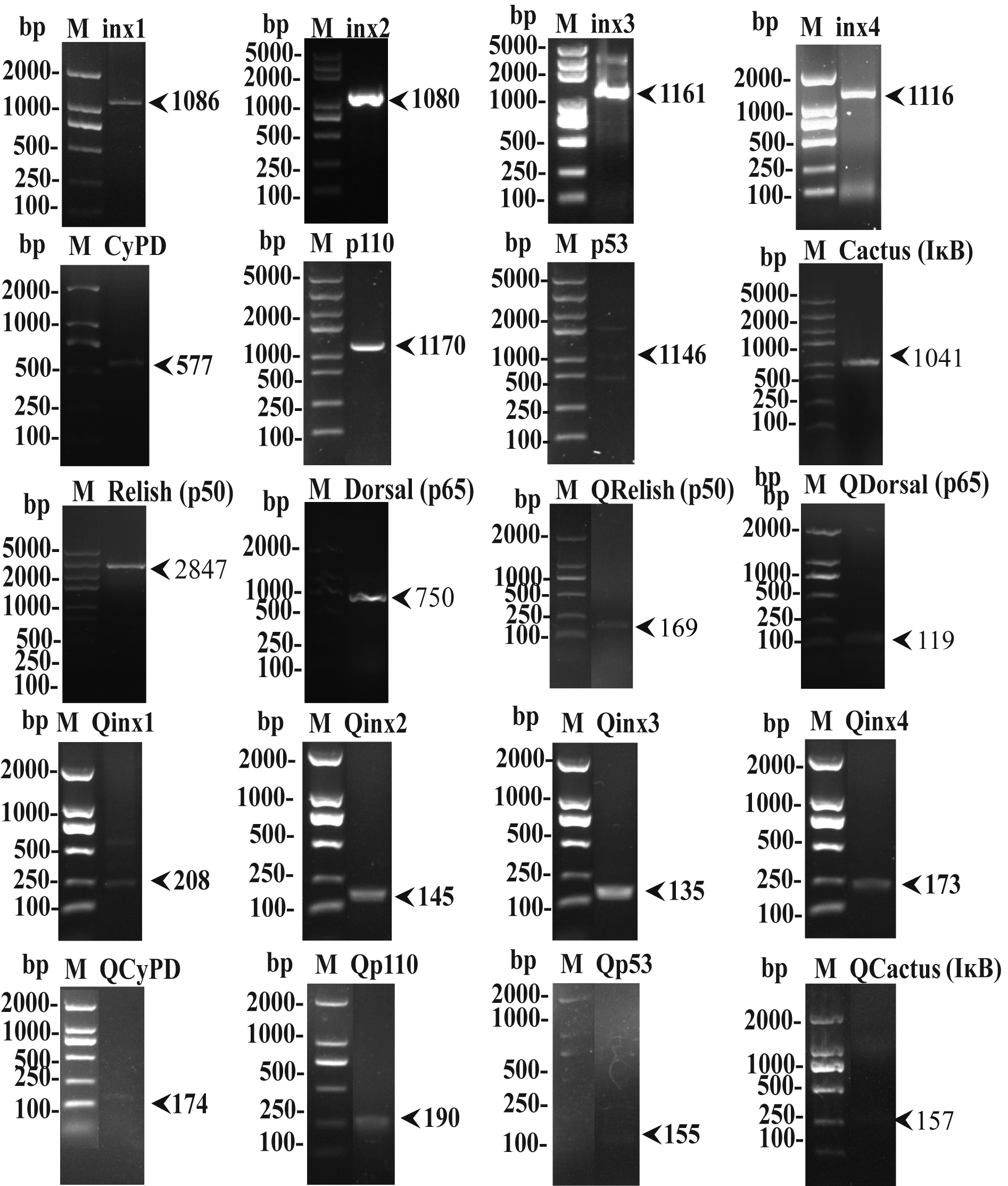

Supplement: Figure S1 — Completed ORF and short qRT-PCR products. (TIF) [file pone.0110967.s001.tif]
